# Serendipity at the Smithsonian: The 107-year journey of *Rhipidocyrtus muiri* Falin & Engel, new genus and species (Ripidiinae, Ripidiini), from jungle beast to valid taxon

**DOI:** 10.3897/zookeys.424.7853

**Published:** 2014-06-08

**Authors:** Zachary H. Falin, Michael S. Engel

**Affiliations:** 1 Division of Entomology, Natural History Museum, 1501 Crestline Drive – Suite 140, University of Kansas, Lawrence, Kansas 66045-4401, USA; 2 Department of Ecology & Evolutionary Biology, University of Kansas, Lawrence, Kansas 66045, USA; 3 Division of Invertebrate Zoology, American Museum of Natural History, Central Park West at 79th Street, New York, New York 10024-5192, USA

**Keywords:** Coleoptera, Ripiphoridae, Smithsonian Institution, taxonomy, morphology, Southeast Asia

## Abstract

The long and tortuous history of an enigmatic and rare new genus and species of ripidiine wedge beetle (Ripiphoridae: Ripidiinae: Ripidiini) from Borneo is discussed and the taxon described and figured as *Rhipidocyrtus muiri* Falin & Engel, **gen. n.** and **sp. n.** The holotype male, and only known specimen, was collected 107 years ago in Borneo but subsequent to this it was transferred among early researchers in the early 1900s. The specimen was dissected and many portions slide mounted, but these were disassociated from the pinned body for more than a generation. A happenstance encounter led to the rediscovery and reassociation of the body and slide-mounted abdomen and other sclerites in 2011, and to its eventual description herein. Ripidiine diversity is briefly discussed and comparisons made between *Rhipidocyrtus* and other members of the subfamily.

## Introduction

Taxa within the ripiphorid tribe Ripidiini are both evolutionarily fascinating and woefully under-described. All members whose biology is known are internal parasitoids of roaches as larvae (Lawrence et al. 2010), a lifestyle likely established at least 90 million years ago resulting in highly derived yet incredibly stable morphologies (Falin and Engel 2010). While the higher level systematics of this lineage have been discussed recently, only a handful of extant species have been named in the last half century (see Falin and Engel in press, and references therein), leaving the true evolutionary breadth and depth of the clade poorly understood. This paper is another small step in the effort to make these rather rare and curious beetles known to science.

**Figures 1–2.**
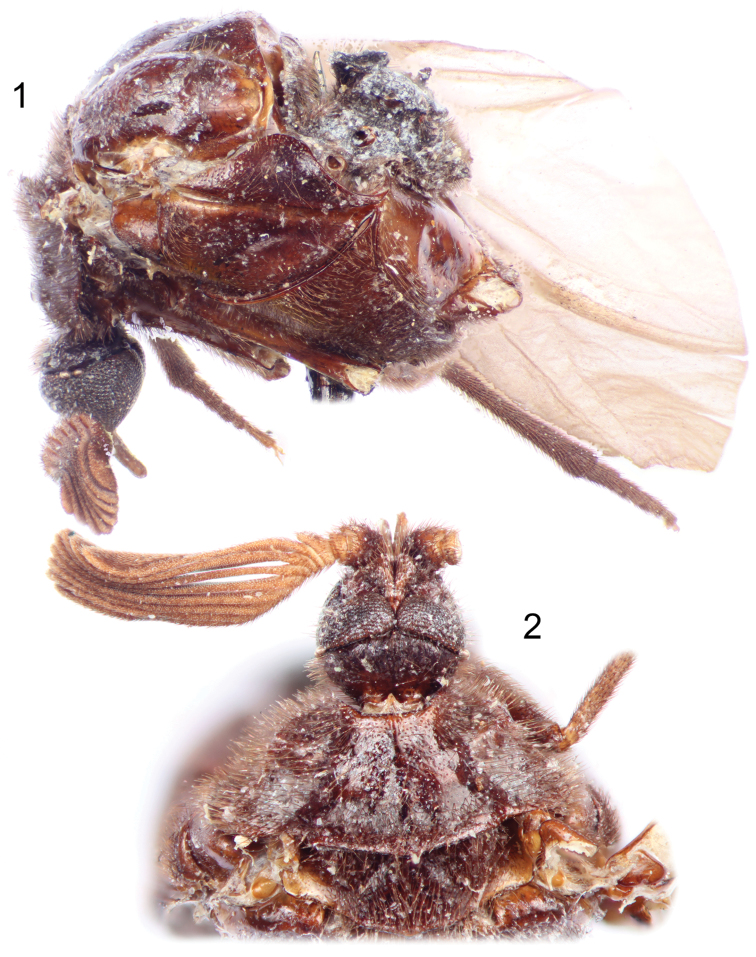
Photographs of holotype male of *Rhipidocyrtus muiri* Falin & Engel, gen. et sp. n. from Borneo. **1** Lateral habitus as preserved **2** Dorsal detail of head and thorax as preserved.

Herein we describe a single new genus and species from West Kalimantan, Borneo, Indonesia, based on a partially disarticulated specimen housed in the Department of Entomology of the National Museum of Natural History (USNM) in Washington, DC (Fig. 1). In this case, despite its striking size, the morphological distinctiveness and phylogenetic importance of the new taxon is debatable. However, the historical aspect of the type specimen itself and how it came to be described is indeed rather remarkable and deserves mention, if only to highlight the role of serendipity (and proper specimen curation) in systematics. The crux of the story takes place in the Casey Room of the USNM in January, 2011, though it begins with Frederick Muir’s travels in Borneo in the summer of 1907.

Although dimly recognized by coleopterists for his collaboration with Dr. David Sharp (1840–1922) on their monographic work on the male genitalia of beetles (Sharp and Muir 1912), Dr. Fredrick A.G. Muir (1872–1931) is perhaps more widely and intimately known for the combination of his pioneer research on biological control agents while employed by the Hawaiian Sugar Planters’ Association (1905–1928) and for his numerous contributions to fulgoroid (Auchenorrhyncha: Fulgoroidea) systematics (Imms 1931, Swezey and Williams 1932). Muir, a quintessential field entomologist, traveled extensively in the Pacific region in the first quarter of the 20^th^ Century; reading accounts of his travels (e.g., Muir 1908) invokes an intense sense of wonder, adventure and nostalgia in all but the most jaded naturalists. One of his epic adventures (in the literal sense) was a 38-month expedition (July, 1906 – Sept., 1909) in search of sugarcane borer biological control agents. This outing led him back and forth from China to Macau, Hong Kong, Singapore and the current nations of Malaysia, Indonesia, and Papua New Guinea, the expedition finally ending in Australia to recover from typhoid fever. No less epic (in the colloquial sense) was Muir’s penchant for taking visitors surfing at Waikiki Beach while visiting the Experimental Station in Hawaii (Paine 1994).

Indeed, it was in the midst of his 1906–1909 expedition, during a six week visit to the island of Borneo (July to September 1907), that Muir collected the specimen that is the subject of this paper. Muir apparently deduced the creature’s parasitic nature and had it sent to William D. Pierce (1881–1967) at the USDA office in Washington, DC. Pierce was obviously familiar with strepsipterans and at least certain ripiphorid clades (Pierce 1904), though his familiarity with the Ripidiini is unclear and he apparently did nothing with the specimen. A terse handwritten note associated with the specimen provides the barest of insights as to its early history while simultaneously revealing Muir’s great interest in it: “This was left with Pierce and after his [Pierce’s] leaving [~1918] Muir visited USNM [~1918] and got [E.A.] Schwarz to find it. Then in 1928 Muir again visited us and called attention of H.S.B. [Herbert S. Barber] [to the specimen] but [Muir] declined to take it back”.

Unlike Pierce, Barber (1882–1950) clearly took interest in the specimen, going so far as to dissect and slide mount portions of the specimen, to create type labels bearing the name proposed here, and providing it with a USNM type number. However, for an unknown reason the nomenclatorial act was never consummated. The slides and pinned specimen became separated (likely after Barber’s death in 1950), the slides curated with the strepsipterans, the pinned specimen with the ripiphorids and the proposed taxon all but forgotten for another 50 years.

The first author (ZHF) visited the USNM in January of 1996, as so many young systematists do, to gather material for what was to become his Ph.D. dissertation. While there, he noticed the majority of the pinned ripidiines, a taxonomically diverse and phylogenetically puzzling lineage within the ripiphorids, had been loaned to John K. Bouseman (1936–2006) of the Illinois Natural History Survey sometime around 1980. Noting the importance of this material to the author’s project and the lack of research done on them in the intervening 16 years, the specimens were eventually transferred to the Snow Entomological Collection at the University of Kansas (SEMC) late in 1997. Like Muir, Barber, and Bouseman, ZHF was struck by the Bornean specimen, though was at the time baffled to find no mention of it in the taxonomic literature.

As it did in Illinois, the specimen remained in Kansas for another 14 years relatively untouched, neither useful for research nor describable as a taxon given what appeared to be its poor state of preservation. However, ZHF visited the USNM again in 2011, coincidentally meeting J. Kathirithamby, a leading expert on Strepsiptera, while there. The two were going about their business in the Casey Room, her looking through slide-mounted strepsipterans, him working on pinned Ripiphoridae when at one point she noted aloud that the three slides in her hand were not strepsipterans at all, but rather ripiphorids and would ZHF care to have a look. He instantly recognized these exquisitely prepared slides as the missing pieces from Muir’s specimen; after well over half a century curated in different parts of the same institution the specimen was once again complete.

Granted, the Casey Room at the USNM is a privileged point of reference; it is not by random chance that entomologists unexpectedly meet there. However, the odds of one of the few leading experts on one obscure taxon recognizing the misidentification of a second obscure taxon and then casually handing slides over the table to one of the few experts on *that* obscure taxon who knows *exactly* what those slides represent is nothing short of statistically incredible. It has taken yet another three years to come to fruition, but Muir’s taxon, so deserving of a name, will finally receive one here, three institutions, at least five systematists, and approximately 107 years after its collection in the mountains of Borneo.

## Material and methods

All observations were made on the single type specimen borrowed from the USNM. This specimen consists of a partially disarticulated adult male (Fig. 1) mounted to an insect pin with a modified minuten, a second insect pin containing ancillary labels, and three microscope slides, all of which are described in detail under the Holotype heading in the species description below.

Measurements were made using an ocular reticle calibrated with a hand-held micrometer observed through and Olympus SZH10 stereomicroscope. Photomicrographs were prepared with a Canon Eos 7D digital camera attached to an Infinity K-2 long-distance microscope lens.

Notal morphological descriptors follow Falin and Engel (in press) and hind wing venation descriptors follow Falin (2003). Other morphological terms used herein (e.g., post-ocular ommatidia) represent a consensus of use by recent authors.

A barcode label bearing the four letter coden for the Snow Entomological Collection (e.g., SEMC1270146) has been added to the pin containing the partially disarticulated specimen. This is explicitly not meant to convey ownership or deposition location of the specimen, it merely allows the taxonomic and collection data of the holotype to be incorporated and served by the University of Kansas’s Division of Entomology’s specimen-level database.

## Systematics

### Family Ripiphoridae Gemminger & Harold, 1870 (1855)
Subfamily Ripidiinae Gerstaecker, 1855
Tribe Ripidiini Gerstaecker, 1855

#### Rhipidocyrtus

Taxon classificationAnimaliaColeopteraRipiphoridae

Falin & Engel gen. n.

http://zoobank.org/1B99831B-55CA-4192-A744-B2D1F5A32F62

##### Type species.

*Rhipidocyrtus muiri* Falin & Engel, sp. n.

##### Diagnosis.

**Figures 3–4. F2:**
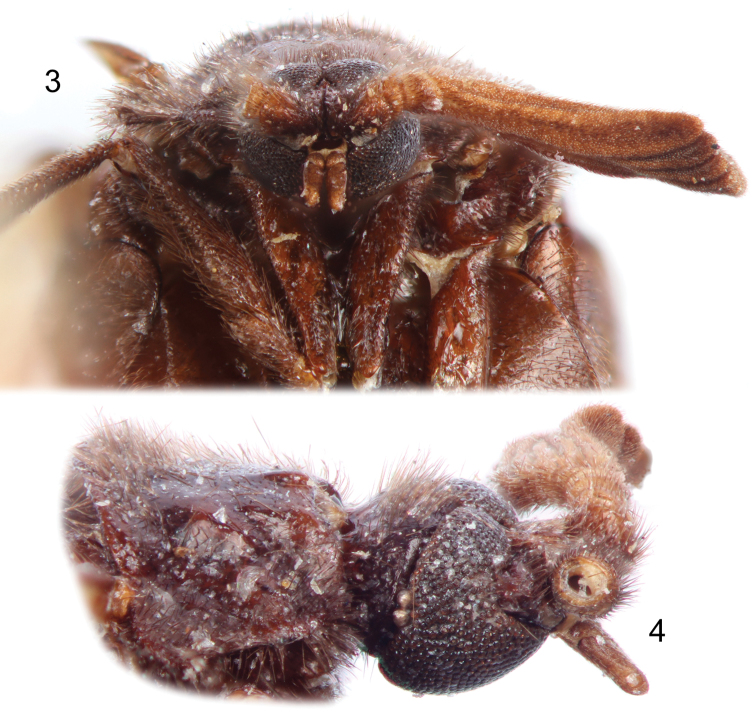
Photographs of holotype male of *Rhipidocyrtus muiri* Falin & Engel, gen. et sp. n. from Borneo. **3** Facial view **4** Right lateral view of head and prothorax.

**Figures 5–6. F3:**
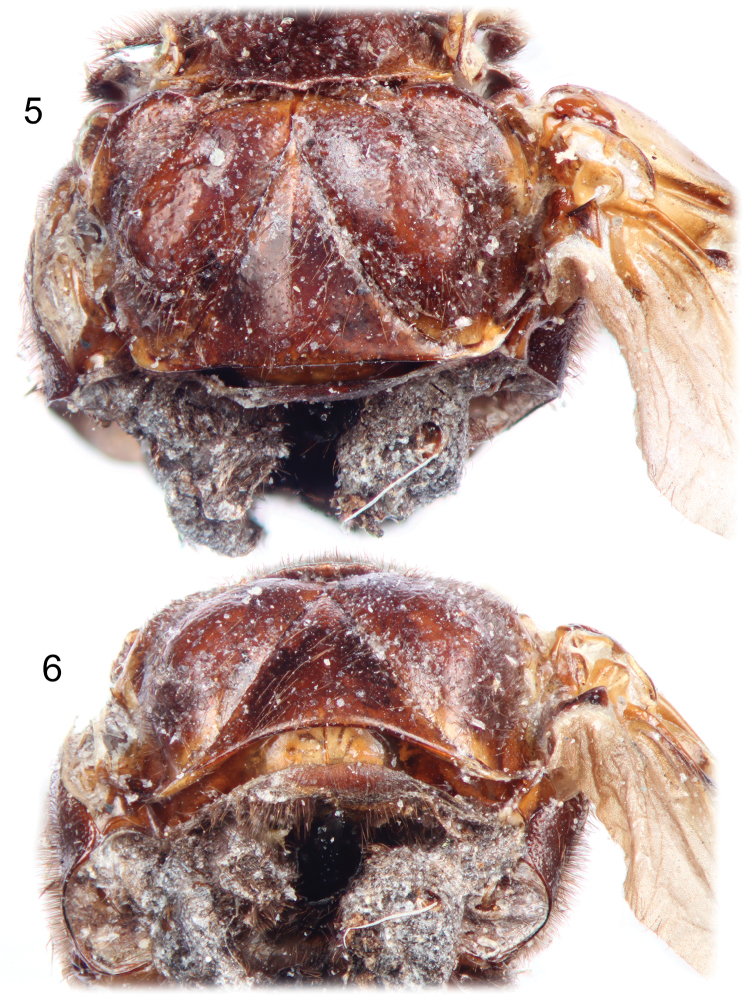
Photographs of holotype male of *Rhipidocyrtus muiri* Falin & Engel, gen. et sp. n. from Borneo. **5** Dorsal detail of metathorax. **6** Posterior view of thorax as preserved.

Closely agreeing with the generalized form of Ripidiini though larger and appearing more hump-backed than typical. *Rhipidocyrtus* possesses the following combination of historically diagnostic characters: unfused, two-segmented maxillary palpi; postocular ommatidia present (Fig. 4); 11 antennomeres, antennomere I simple (Figs 2, 3), antennomere II toroidal, antennomere III more robust, produced medially (Fig. 2), antennomeres IV–XI strongly uniflabellate (Figs 2, 9); mesoscutellum present but weakly developed (Fig. 2), posterior margin very weakly bisinuate with a small medial point (Fig. 5); tarsal formula 5-5-4 (Figs 10–12). *Rhipidocyrtus* differs from all known ripidiines in the form of the metanotum (Figs 5, 6), possessing what appears to be a reduced “metascutellar box” visible at the anterior margin of the metanotum to either side of the midline. This structure is typically either robust (most New World taxa) or absent (most Old World taxa). Likewise, *Rhipidocyrtus* appears unique in that the metascutellum narrows evenly to an anterior point, terminating at the anterior margin of the metanotum as a single medial sulcus (Fig. 5) (but see the following comparative comments).

##### Etymology.

The new genus-group name is one of several spelling variants first composed and applied to the holotype specimen and slide labels by H.S. Barber. It is a combination of the Greek words *rhipido*, meaning, “fanlike”, and *cyrtus*, meaning, “curved”. Our interpretation is that it is meant to describe the unusually convex or “hump-backed” appearance of the type species. The name is masculine.

##### Comments.

*Rhipidocyrtus* is superficially distinctive within the Ripidiini for its particularly hump-backed facies, its relative size (it is the largest ripidiine known), and possessing a pronotum that is strongly dorso-ventrally compressed laterally, resulting in an unusually abrupt lateral margin. However, despite this novel first impression it is in most regards morphologically unremarkable. It shares with most other Old World taxa a well-developed and medially-produced antennomere III as well as the presence of a mesoscutellum (albeit weakly produced), suggesting a strong relationship with that putative lineage [see Batelka et al. (2011) and Falin and Engel (in press) for discussions of supra-generic character patterns within the Ripidiini].

That said, *Rhipidocyrtus* exhibits what is here tentatively described as a rudimentary “metascutellar box” *sensu* Falin and Engel (in press). Apart from the Old World genus described in that paper, this structure is exclusive to New World ripidiines; finding an otherwise typical Old World taxon with a rudimentary “box” may prove phylogenetically significant. However, the homologous nature of this “box” remains poorly understood and, in the case of *Rhipidocyrtus*, this structure may simply be an artefact of the relatively more developed flight musculature necessary for such a large individual.

While likely less phylogenetically significant than the “metascutellar box”, the form of the metascutellum is considerably more obvious and also serves to differentiate this taxon from its close relatives. Typically, the metascutellum narrows anteriorly, its margins becoming more or less parallel as they terminate at either the anterior margin of the metanotum or at the metascutellar box, depending on the lineage. In the case of *Rhipidocyrtus*, the lateral margins converge to form a single median sulcus well before the anterior metanotal margin. A similar arrangement is illustrated for the fossil genus *Paurorhipidius* Kaupp and Nagel, though the authors state the metanotal structures are obscured in the type specimens (Kaupp et al. 2001) and the exact configuration is indeed uncertain. Likewise, species of the genus *Blattivorus* Chobaut tend to have anteriorly-narrowed, parallel-sided metascutella, in some cases terminating just before the anterior metanotal margin. However, *Blattivorus* appears to be a well-defined monophyletic lineage not closely related to *Rhipidocyrtus*; it is unlikely the superficially similar forms of the metascutellur apex are truly homologous.

#### Rhipidocyrtus muiri

Taxon classificationAnimaliaColeopteraRipiphoridae

Falin & Engel sp. n.

http://zoobank.org/FCE0859D-3AF2-42DC-BCBC-1D915779FD9D

Figs 1 [Fig F2] [Fig F3] [Fig F4] –15

##### Holotype.

♂, USNM Type No. 41869, Department of Entomology, US National Museum of Natural History, Smithsonian Institution, Washington DC, USA; partially disarticulated, specimen preparation and labels distributed on two pins and three microscope slides as follows: first pin has only the original specimen labels; second pin contains the complete head, thorax, and poorly preserved portions of abdominal segments I–III mounted on a modified minuten (Fig. 1), the right elytron is glued to the minuten; first slide has the right antenna and left middle and hind legs; second slide has the left elytron, hind wing, and foreleg; third slide has the splayed abdomen and genitalia. Specimen labels read as follows [each preparation starts as a distinct paragraph, different lines of those labels separated by a slash (/) and separate labels by double slashes (//)]:

Pin 1: No specimen parts: “Borneo / 383” // “Rhipideus [sic]” // “This was left with Pierce / and after his leaving / Muir visited USNM / and got Schwarz to find / it. Then in 1928 Muir again / contacted us & called attention / of H.S.B. but declined to take it back.” [underside of last label reads] “Found by / F. Muir / on flowers / P.T.O.” // “HOLOTYPE / Rhipidocyrtus / muiri / Z.H. Falin & M.S. Engel”.

Pin 2: Head, thorax, + basal abdominal segments, right elytron glued to minuten: “F. Muir #383 / on flowers / Aug 1907 / Mowong / Borneo” // “Ripidius / muiri Bar. / U.S.N.M. / Type no. / 41869” // “SEMC1158329 / KUNHM-ENT” // “HOLOTYPE / Rhipidocyrtus / muiri / Z.H. Falin & M.S. Engel”.

Slide 1: “muiri / Barber / antenna, middle & hind leg / F. Muir #383 on flowers / Aug 1907 / Mowong Borneo. / Holotype No. 41869 U.S.N.M.”.

Slide 2: “Rhipidocyrtus / muiri / Barber / left wing elytron / & front leg / F. Muir #383 on flowers / Aug 1907 Mwong Borneo / Type No. 41869 U.S.N.M.”.

Slide 3: “Rhipidocyrtus / muiri / Barber / ♂ genitalia / & abd. seg. / spiracles nos. 3 (front) / 4, 5, & 6. – Mwong Borneo / F. Muir / Type No. 41869 U.S.N.M.”.

##### Diagnosis.

As per the generic diagnosis above.

##### Description.

**Figures 7–12. F4:**
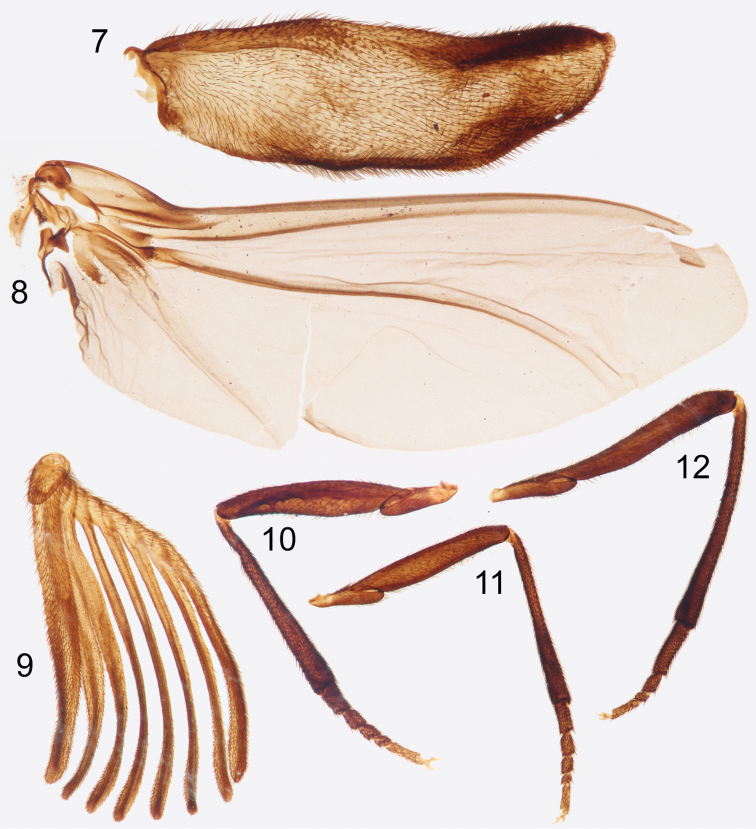
Photographs of slide mounted structures from holotype male of *Rhipidocyrtus muiri* Falin & Engel, gen. et sp. n. **7** Elytron **8** Hind wing **9** Right antenna **10** Foreleg **11** Mid-leg **12** Hind leg.

**Male.** Large, though difficult to measure given the longitudinally arched facies and the partial disarticulation; approximately 2.8 mm long in dorsal view from anterior margin of pronotum to posterior margin of metascutellum, approximately 2.2 mm wide at base of pronotum (although a gross approximation given the disarticulation involved, total length in life might approximate 6 mm); elytron length 2.4 mm; hind wing length 6.5 mm. Body nearly unicolorous brown (Figs 1–6); antennomere I similar in color to body, antennomeres II–XI lighter brown (Figs 1, 2) as are various subregions of the notum (e.g., posterolateral mesonotal angles). Elytra coriaceous, more or less translucent brown, darker along margins and with short, suberect setae (Fig. 7). Hind wing typical, very lightly pigmented at most (Fig. 8), but covered with microsetae giving an infuscate appearance and a reflective sheen.

Head subspherical (Figs 2, 4), slightly compressed dorso-ventrally (Fig. 3). Vertex weakly convex (Fig. 4), sloping uniformly to occiput, integument shining with indistinct punctation and weak, irregular, sculpturing. Dorsal and ventral aspects of head with suberect to erect setae. Compound eyes large (Figs 3, 4), coarsely faceted, with erect setae dorsally; weakly convergent dorsally, strongly convergent ventrally, occupying nearly the entire ventral surface of the head (Fig. 3); two large post-ocular ommatidia present at posterolateral margins of compound eyes (Fig. 4). Frons obsolete between antennal bases and maxillary palpi, these structures dorsoventrally contiguous (Fig. 3). Maxillary palpi two-segmented, basal segments free, obliquely toroidal, apical segments approximately 3.5 to 4 × length of basal segments (Figs 3, 4), fusiform, broadest near base, with subapical, obliquely-depressed sensory pits.

Antennae consisting of 11 antennomeres; antennomere I stout (Fig. 3), asymmetrically cup-like, apical opening produced laterally; antennomere II irregularly toroidal, longest at midline, subequal to III; antennomere III similar in shape to II except strongly produced mesally (Fig. 2); antennomeres IV–X with mesally facing rami (Figs 2, 9), bases of IV–VI longitudinally compressed, subequal, base of antennomere VII approximately 2 × length of antennomere VI; antennomere XI expanded, similar in shape to rami of previous segments (Fig. 9); antennae constructed such that rami VIII and IX nearly equal in apparent length, rami decreasing subequally in apparent length to either side, rami V shortest in apparent length; antennomeres I and II with moderately dense suberect setae similar to those of head, similar setae present on bases of antennomeres III–X but diminish in length apically; rami of antennomeres with specialized sensory trichia beginning with mesal projection of antennomere III.

Pronotum with suberect to erect setae, integument shining, weakly, irregularly punctate; pronotum broadly bell-shaped in dorsal view (Fig. 2); anterior margin broadly excavate; anterolateral angles rounded, nearly obsolete, strongly deflected ventrally; posterior margin gently arcuate with a small medial projection, deflected dorsally (Fig. 1); posterolateral angles broadly rounded, projecting, slightly concave on surface and deflected dorsally; lateral margins evenly arcuate, converging anteriorly, strongly dorso-ventrally compressed, proplurae reduced, hidden in dorsal view. Pronotal disc with a raised medial tubercle at anterior margin and a weakly rounded medial carina extending posteriorly approximately ¼ length of pronotum, gradually becoming obsolete, otherwise disc gently but irregularly convex laterally with two large but weak convexities near lateral margins, apparently demarcating internal articulation points of procoxae.

Mesonotum with suberect, posteriorly-facing setae, integument shining, very weakly punctate, appearing nearly glabrous; posterior margin weakly bisinuate with medial projection (Fig. 5), forming a broad but narrow mesoscutellum; posterolateral angles obtusely rounded, deflected dorsally; lateral and anterior margins obscured. Mesonotal disc gently but irregularly convex laterally (Fig. 2), with a large convexity on either side of midline near anterior margin.

Metanotum with scattered recumbent to suberect setae (many appear abraded on holotype specimen), integument shining, punctation variably weak and scattered to nearly obsolete with exception of metapostscutellum described below. Metascutum apparently divided into three regions – anteromedial box, anterolateral lobes, and posterolateral lobes (Figs 5, 6). Anteromedial box partially obscured medially by mesocutellum but appears to form a contiguous, narrow band separated posteriorly from posterolateral lobes by an arcuate impressed sulcus and laterally from anterolateral lobes by indistinctly impressed longitudinal constrictions. Anterolateral lobes obliquely convex and themselves separated from obliquely convex posterolateral lobes by wide, deep and relatively setose impressions (Fig. 5). Metascutellum clearly delineated by a pair of oblique, deeply impressed sulci curved basally (Figs 5, 6), nearly linear anteriorly, converging to a single impressed medial sulcus terminating at apparent anterior metanotal margin (Fig. 5). Posterior margin of metascutellum straight in dorsal view (Fig. 5), gently convex dorsoventrally; metascutellar disc gently convex (Fig. 6) with a weak, rounded carina originating at apex, continuing approximately 1/3 length of metascutellum (Fig. 5), gradually becoming obsolete. Metapostscutellum a relatively narrow band positioned more or less dorso-ventrally, ventral and slightly anterior to posterior margin of metascutellum (Fig. 6), posterior margin of metapostscutellum strongly deflected dorsally (Figs 1, 6). Surface of metapostscutellum glabrous, impunctate except posterior-facing aspect of posterior marginal flange appearing setose due to superimposed abdominal tergite I.

Lateral and ventral aspects of pterothorax typical of tribe, if slightly exaggerated in form; vestiture and texture similar to notum, setae more or less uniform, suberect; punctation variable, generally scattered and weak to nearly obsolete except as noted. Mesepisternum fused with mesosternum; mesepimeron a prominent, rounded flange separated from mesepisternum by a strong invagination. Metepisternum typical, dorsoanterior lobe present, nearly glabrous and impunctate (Fig. 1). Metepimeron separated from metepisternum by a strongly invaginated sulcus (Fig. 1), strongly dorsally arcuate in lateral view, widest near middle, tapering evenly to a point anteriorly, tapering posteriorly as well but then slightly thickening and recurving posteriorly.

Legs typical; coxae, trochanters, and femora smooth, shining with suberect setae and scattered punctation. Tibiae clothed in more stout, spine-like setae, punctation much closer, integument appearing nearly granular; tibiae more or less straight (Figs 10–12), cylindrical, broadening slightly apically; apical spurs absent. Tarsi 5-5-4 (Figs 10–12), setation and texture similar to tibiae; all tarsomeres more or less cylindrical, progressively subequal in diameter, and obliquely truncate apically; apical tarsomeres obliquely tapered basally; protarsomere I approximately 1.5 × length of protarsomere II, protarsomeres II and III subequal, protarsomere IV approximately 0.5 × length of protarsomere III, protarsomere V approximately equal to protarsomeres II and III combined; length of mesotarsi greater than that of protarsi, but relative ratios similar. Metatarsomere I approximately as long as metatarsomeres II–IV combined, metatarsomere II 2 × length of metatarsomere III, metatarsomere IV approximately equal to metatarsomeres II and III combined. Pretarsal claws small, simple, sickle-shaped.

Elytra as described above; widely separated, short, both disarticulated in holotype but approximately extending just past posterior margin of metanotum when closed. Deformed in preservation, lateral margin somewhat thickened (Fig. 7), both margins widening slightly in basal 1/3, roughly parallel in medial 1/3, then medial margin tapering unevenly laterally in apical 1/3, forming a blunt, rounded apex nearest lateral margin. Hind wing also as above, with vein R parallel to and more or less fused with C+Sc, terminating prior to wing apex (Fig. 8); vein Cu well defined, 2^nd^A_3_+3^rd^A_1_ less so, each reaching wing margin (Fig. 8).

**Figures 13–15.**
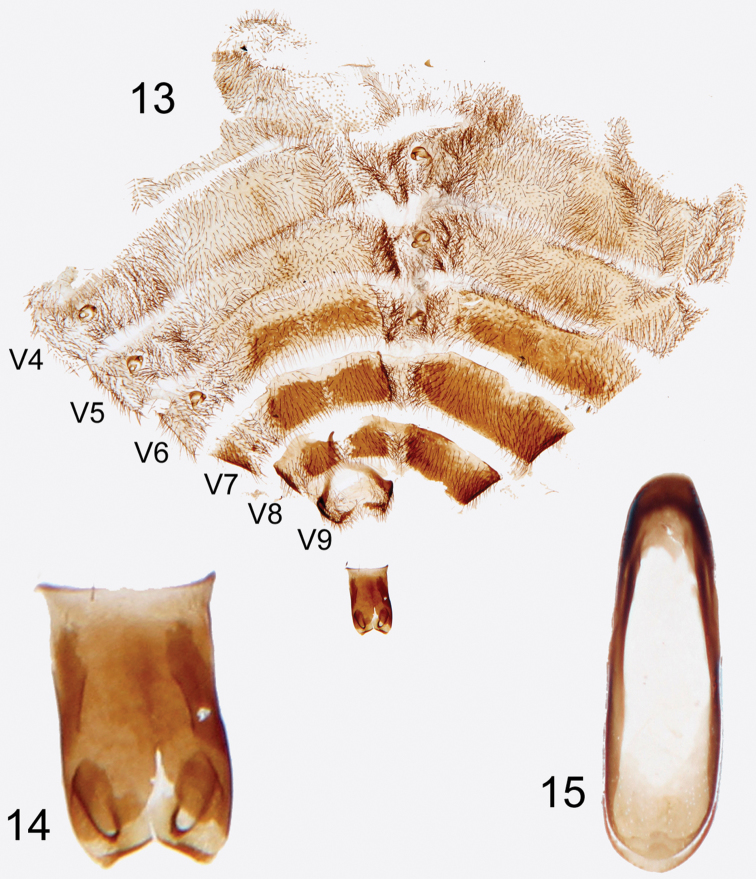
Photographs of slide mounted abdominal structures from holotype male of *Rhipidocyrtus muiri* Falin & Engel, gen. et sp. n. **13** Splayed abdomen as preserved on slide, numbered ventrites to the left, unnumbered tergites to the right **14** Enlarged detail, ventral view of tegmen **15** Enlarged detail of median lobe.

Abdomen partially disarticulated in holotype specimen making *in situ* characterization difficult. Abdomen likely bluntly sub-conical, possibly dorso-ventrally compressed in life; with eight (I–VIII) visible tergites and seven (II–VIII) visible ventrites; well-sclerotized spiracles present in poorly-defined pleural region of segments I–VI (Fig. 13); tergites and ventrites fairly uniformly setose (Fig. 13), pleural regions slightly more densely. Tergites I–V and ventrites II–V weakly sclerotized; remaining visible abdominal segments (VI–VIII) comparatively more so (Fig. 13), color similar to that of body, integument virtually impunctate. Abdominal segment IX with dorso-posterior margin evenly emarginate though sclerotization gives it a bilobed appearance (Fig. 13); dorso-ventrally convex, lobes fusing ventrally, forming a spine projecting anteriorly and asymmetrically to left in dorsal view (Fig. 13).

Tegmen appearing typical for tribe (Fig. 14); an approximately bilaterally symmetrical tube sclerotized dorsally, open ventrally, truncate but slightly flared basally, deeply emarginate apically. Gonoforceps similarly sclerotized, membranously articulated to apex of tegmen dorsally, lateral articulation difficult to discern, also more or less bilaterally symmetrical and consisting of paired, medio-obliquely truncate lobes dorsally and medio-obliquely oriented digitiform projections ventrally. Median lobe extremely simple, essentially appearing as a strongly beveled ovoid ring, the basal end with a dorsally sclerotized shelf and the apical end with a ventrally sclerotized shelf (Fig. 15).

**Female.** Unknown.

##### Immature stages.

Unknown.

##### Etymology.

The specific epithet is as proposed by H.S. Barber and meant to commemorate Dr. Frederick Muir, a remarkable and inspiring entomologist.

##### Comments.

Most ripidiine species, this one included, are described on the basis of very few, if not unique specimens, naturally making estimations of intra-specific variation in size and appearance difficult. In the few cases in which we have examined long series of a single species, such variability appears to be quite low. We expect, then, that additional specimens of *Rhipidocyrtus muiri* will hew quite closely to the above description.

## Discussion

As currently understood, Southeast Asia is home to two precinctive ripidiine genera (e.g., *Falsorhipidius* Pic and *Pseudorhipidius* Chobaut) containing three species in total. Four additional nominal species in the widespread Old World genus *Ripidius* Thunberg have been described from Southeast Asia, though their taxonomic placement and status is uncertain. At least five new species spanning two established genera (e.g., *Pseudorhipidius* and the Australian genus *Rhipidioides* Riek) and two new genera, one precinctive to Southeast Asia, one not, await description (Falin and Engel in press, unpublished data). Undoubtedly additional specimens representing additional new taxa reside in the world’s collections; the total number of ripidiine taxa that may yet be documented from this under-collected yet critically threatened region is sobering indeed.

While Southeast Asia stands out as an area of high diversity and endemism for the tribe, only one other species, *Ripidius angusticollis* Pic, 1943, has been described from Borneo (in Pic’s infamously succinct and uninformative style). Notes taken during a cursory examination of the putative type by ZHF during a 1996 visit to the Muséum National d’Histoire Naturelle, Paris, provide no evidence to contradict its placement within *Ripidius* and certainly foreshadow no close relationship with *Rhipidocyrtus*.

Uncertainty remains as to the exact type locality for *Rhipidocyrtus muiri*, the house and lands associated with a mining concession owned by an Englishman named Mr. Girdlestone. It is variously transliterated as “Mwong”, “Mowong”, and in Muir’s own accounts “Moewong” (Muir 1908), though does not appear to correspond to any similarly named extant locality today. Muir spent “two weeks” there, from 10 August to approximately 24 August 1907 and “would willingly have spent two years” (Muir 1908: 56). While this has been cited as the type locality for numerous new taxa (e.g., Muir 1913, 1923; Carvalho 1983), it does not appear to have been definitively pinpointed in the literature. A close reading of Muir’s travel notes suggests Moewong may be the elevated point at 0.7621°N, 109.4298°E, approximately 2 km SSW of the settlement currently called Tirta Kencana, Bengkayang District, Bengkayang Regency, West Kalimantan, Indonesia. A more thorough investigation of Muir’s collecting localities would be both fascinating and scientifically profitable.

Apart from the notal characters tentatively described above, little about the morphology of *Rhipidocyrtus* lends itself to strong phylogenetic inference. It remains to be seen whether the genus falls neatly within the standard Old World ripidiine lineage as current evidence suggests or perhaps just outside the clade, in some way intermediate between the Old and New World morphological archetypes.

Likewise, the relative lack of complexity of the male genitalia despite the size of the specimen and the exquisite preparation is somewhat disappointing, indicating that male genitalia may be generally uninformative at the species level and perhaps even among closely related genera within the tribe. Little comparative work has been done on ripiphorid genitalia (see Rivnay 1929, Selander 1957; genitalic comparisons were not attempted by Falin 2003); our knowledge of the subject remains distressingly fractured and incomplete, particularly in regards to the Ripidiinae. Besuchet’s (1956) morphological examination of *Ripidius quadriceps* Abeille, 1872, stands as both the best and only detailed study within the subfamily. Although a detailed comparison of male ripidiine genitalia falls outside the scope of this paper, it is interesting to note that while similar in overall structure, differences in the postero-dorsal margins of abdominal segment IX and the tegmen, the appearance and relative sclerotization of the parameres and, while simple, the form of the median lobe appears to differ between *Ripidius quadriceps* and *Rhipidocyrtus muiri*; these and other genitalic characters may prove useful in future comparisons.

Lastly, it is worth considering again the circuitous and serendipitous path this specimen took to description. While lapses in personal scientific productivity are common, indeed inevitable, they can eventually be overcome with good personal and institutional specimen curation. We will never know why Barber failed to follow through with the original description despite his obvious interest and efforts. However, we do know that the specimen and its component slides were separated, either by Barber himself or, more likely, by some harried staff member preparing his office for its next occupant. It took well over half a century for an incredibly unlikely meeting of systematists to occur to transcend that particular curatorial oversight. Thankfully, the specimen will return to Washington, DC, “whole” and validly named.

## Supplementary Material

XML Treatment for Rhipidocyrtus

XML Treatment for Rhipidocyrtus muiri
